# The Obesogenic Quality of the Home Environment: Associations with Diet, Physical Activity, TV Viewing, and BMI in Preschool Children

**DOI:** 10.1371/journal.pone.0134490

**Published:** 2015-08-06

**Authors:** Stephanie Schrempft, Cornelia H. M. van Jaarsveld, Abigail Fisher, Jane Wardle

**Affiliations:** 1 Health Behaviour Research Centre, Department of Epidemiology and Public Health, University College London, London, United Kingdom; 2 Department for Health Evidence & Department of Primary and Community Care, Radboud University Medical Center, Nijmegen, The Netherlands; Institute of Preventive Medicine, DENMARK

## Abstract

**Objectives:**

The home environment is thought to play a key role in early weight trajectories, although direct evidence is limited. There is general agreement that multiple factors exert small individual effects on weight-related outcomes, so use of composite measures could demonstrate stronger effects. This study therefore examined whether composite measures reflecting the ‘obesogenic’ home environment are associated with diet, physical activity, TV viewing, and BMI in preschool children.

**Methods:**

Families from the Gemini cohort (n = 1096) completed a telephone interview (Home Environment Interview; HEI) when their children were 4 years old. Diet, physical activity, and TV viewing were reported at interview. Child height and weight measurements were taken by the parents (using standard scales and height charts) and reported at interview. Responses to the HEI were standardized and summed to create four composite scores representing the food (sum of 21 variables), activity (sum of 6 variables), media (sum of 5 variables), and overall (food composite/21 + activity composite/6 + media composite/5) home environments. These were categorized into ‘obesogenic risk’ tertiles.

**Results:**

Children in ‘higher-risk’ food environments consumed less fruit (OR; 95% CI = 0.39; 0.27–0.57) and vegetables (0.47; 0.34–0.64), and more energy-dense snacks (3.48; 2.16–5.62) and sweetened drinks (3.49; 2.10–5.81) than children in ‘lower-risk’ food environments. Children in ‘higher-risk’ activity environments were less physically active (0.43; 0.32–0.59) than children in ‘lower-risk’ activity environments. Children in ‘higher-risk’ media environments watched more TV (3.51; 2.48–4.96) than children in ‘lower-risk’ media environments. Neither the individual nor the overall composite measures were associated with BMI.

**Conclusions:**

Composite measures of the obesogenic home environment were associated as expected with diet, physical activity, and TV viewing. Associations with BMI were not apparent at this age.

## Introduction

High rates of overweight and obesity[1] have prompted research into prevention. The preschool period has been identified as a critical time for the development of overweight and obesity[2], with evidence that many young children engage in behaviors that promote excess weight gain[3,4]. The home environment is thought to be particularly influential in establishing early weight trajectories, providing an avenue for long-term obesity prevention[5,6].

**Fig 1:**
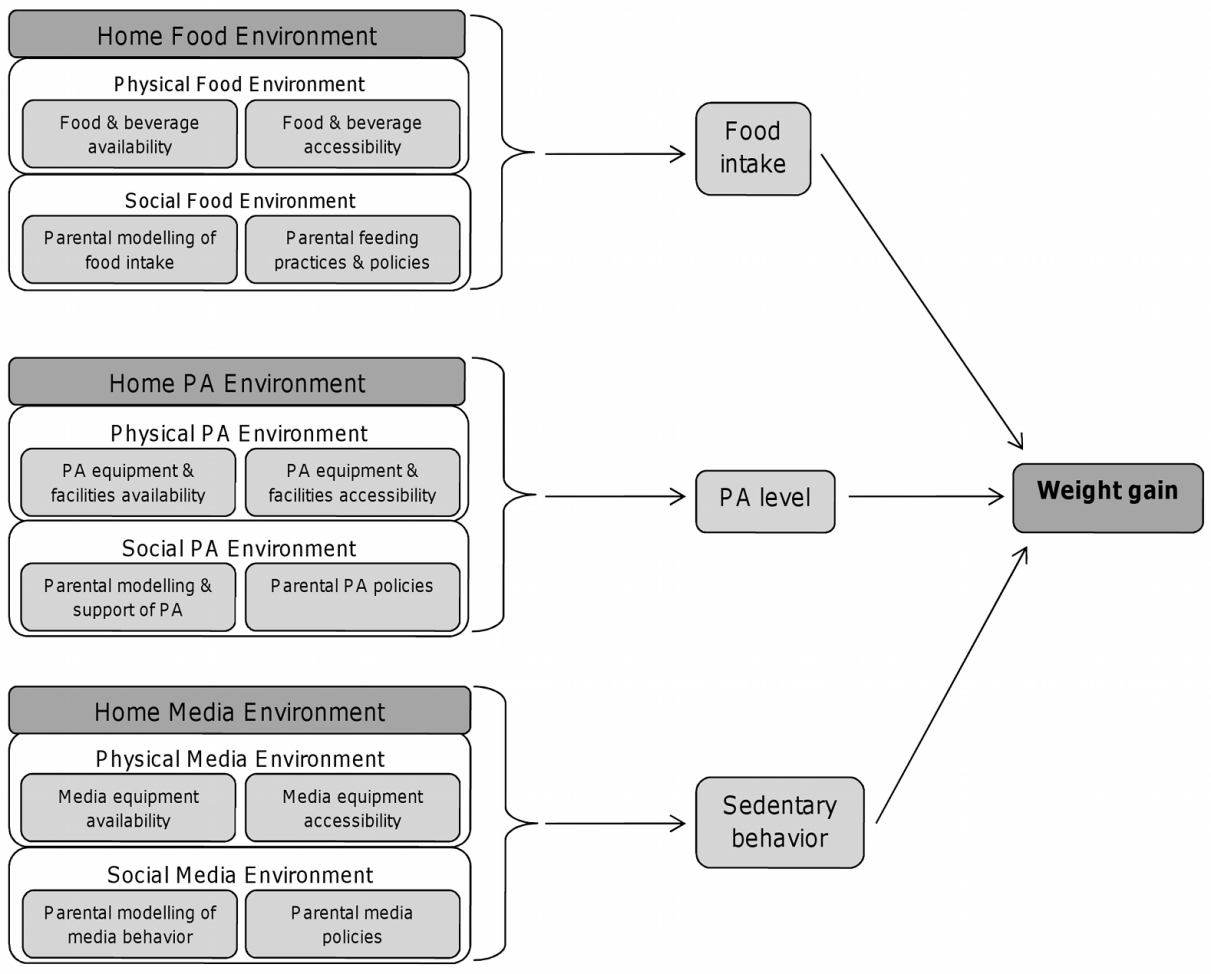
Simple conceptual model of home environment influences on diet, physical activity, sedentary behaviors, and weight (adapted from Gatshall et al, 2008[8]). PA = physical activity.

The ‘obesogenic’ home environment has been defined in terms of food and activity-related domains[7,8]. Each domain comprises a number of physical and social aspects that are hypothesized to influence corresponding diet, physical activity, and sedentary behaviors, and cumulatively weight (see **Fig 1**). A number of studies have examined associations between aspects of the home environment and diet, physical activity, and sedentary behaviors in preschool children[9]. Comparatively fewer studies have examined associations between the home environment and weight, particularly in preschool children. Some larger-scale studies have found that having a TV in the child’s bedroom[10] and family meals[11] are associated with weight in this age group.

Given that any one aspect of the home environment probably has limited influence on weight-related outcomes, composite indicators incorporating all domains should capture the overall level of risk for weight gain more effectively. This is also important because different aspects of the home environment may enhance or counteract one another. For example, a home may have much media equipment but also be supportive of physical activity. One study found that total scores on the Family Nutrition and Physical Activity screening tool were associated with one-year BMI change, after adjusting for baseline BMI, parental BMI, and other demographic factors, in a sample of 6 to 7-year-olds (n = 1030)[12]. The total score is the sum of items assessing parental modelling of nutrition and physical activity, parental monitoring of screen time, parental use of food restriction and reward, and family meals, which were constructs identified by an evidence analysis project in America. However, the total score also incorporates the child’s diet, physical activity, screen time, and sleep so the influence of the home environment per se was not estimated.

More recently, two studies used pattern analytic techniques to create composite measures of household obesogenic risk in adolescents[13,14]. Grunseit and colleagues used Principal Component Analysis to determine the collective influence of 11 home environment constructs on adolescent diet, physical activity, and screen time. Parental confidence about their child’s soft drink intake, confidence about their child’s physical activity participation, having rules around TV viewing, offering their child water to drink with meals, and frequency of child eating breakfast loaded onto the first factor, labelled ‘obesogenic control’. Soft drink availability at home, having a TV in the child’s bedroom, family frequency of going to fast food outlets, frequency of child eating dinner in front of the TV, and number of short car trips loaded onto the second factor, labelled ‘obesogenic risk’. ‘Obesogenic control’ was associated with higher intake of healthy foods, lower intake of unhealthy foods, higher physical activity, and less screen time, while ‘obesogenic risk’ was associated with lower intake of healthy foods, higher intake of unhealthy foods, lower physical activity, and more screen time[13]. The second study used a cluster analytic technique to identify specific family ‘types’ associated with parent and adolescent BMI. Both parent and adolescent BMI were highest in ‘unenriched/obesogenic’ families, characterized by higher levels of parental depressive symptoms, higher levels of parental screen time, fewer family rules around meal time, less physical activity equipment, and lower variety of foods in the home than ‘Risky Consumer’ and ‘Healthy Consumer/Salutogenic’ families[14].

Although these studies are important, using pattern analytic techniques to derive a composite score is problematic as some variables may not load onto the latent factor(s) even though they are relevant to weight. Indeed, in the study by Grunseit and colleagues, parental use of sweets to reward behavior (which has been associated with increased consumption of energy-dense foods and beverages[15]), was removed from the analysis as it did not load onto the latent factors. It is not necessarily expected that independent risk factors for obesity will be related even if each is relevant to weight.

Two comprehensive measures of the home environment have recently been developed: the Healthy Home Survey[16] and the Comprehensive Home Environment Survey[7]. Both have good reliability and validity, and the Comprehensive Home Environment Survey (not available at the time of this study) has a procedure for generating a total score (the subscales are rescaled to range from 0 to 1 and then summed; with higher scores representing ‘healthier’ homes). Total scores were correlated with lower BMI in a low-income US sample of 5 to 17-year-olds; although analyses were not adjusted for covariates[7]. No studies have used comprehensive measures in a composite form to examine associations with weight in preschool children.

The aims of the present study were therefore to examine associations between composite indicators of the ‘obesogenic’ home environment and (i) diet, physical activity, and TV viewing, which are assumed to be determined by the environment, and (ii) bodyweight, which is assumed to be a consequence of a sustained positive energy balance, in a sample of preschool children.

## Methods

### Participants and study design

Data were from parent-child dyads in the Gemini twin birth cohort (one child randomly selected from each twin pair to avoid clustering effects). Gemini was set up in 2007 to examine environmental and genetic influences on early weight trajectories, and has been described in detail elsewhere[17]. Briefly, 2402 families (36% of all live twin births in England and Wales during the recruitment period March–December 2007) consented to participate and completed a baseline questionnaire when their children were on average 8.2 months old (SD = 2.2, range = 4–20 months). The Home Environment Interview (HEI) was completed by 1113 families (46% of the total sample) when the children were on average 4.2 years old (SD = 0.4, range = 3–5 years). The study sample (n = 1096) comprised families with data on all variables included in the analysis.

### Ethics statement

Parents provided written informed consent for their family to participate in the study. Ethical approval was granted by the University College London Committee for the Ethics of non-National Health Service Human Research. All aspects of data collection and storage were in compliance with the standards specified by this body.

### The Home Environment Interview

The HEI was adapted from the Healthy Home Survey, which was the most comprehensive measure of the home environment available at the time and had been used with parents of young children[16]. The Healthy Home Survey assesses a range of physical and social aspects of the home food, activity, and media environments. Amendments were made to make the language UK-specific, and additional scales were included to assess parental support of physical activity[18], parental TV viewing[19], and neighbourhood satisfaction[20]. Parental feeding practices were examined using validated questionnaires[21–24]. The HEI was administered as a computer-assisted telephone interview by trained researchers; it took around 30 minutes (see S1 File). A sample of 44 mothers completed a second telephone interview 7–19 days later (mean = 9.6, SD = 3.4) to assess test-retest reliability of the measure.

Only constructs generally agreed to be relevant to weight gain were included in the home environment composite scores. Relevant constructs were identified using a review of the literature, and corroborated by a panel of 30 experts in the child obesity field. Expert consensus was obtained using a single-round Delphi method[25]. The experts were identified through an internet search or were already known to have expertise in the field, and were contacted by email. The email contained a link to an online survey, which presented the list of home environment variables and asked the experts to indicate whether or not they thought each one was associated with increased or decreased risk for weight gain in childhood. The survey was completed anonymously and the consensus conclusions were emailed to all those initially contacted.

A total of 32 home environment constructs were included in the composites. These are presented in **Table 1**, along with descriptive statistics. The correlations between the home environment variables in each domain were mostly small (more than 75% of the correlations within each domain were 0.10 to 0.20, or less than 0.10). Six (9.5%) of the home food environment correlations, 1 (6.7%) of the home activity environment correlations, and 2 (20%) of the home media environment correlations were greater than 0.30. There were three large correlations (≥0.50) between the following variables: parental monitoring and covert restriction (0.53), parental monitoring and restriction (0.53), and respondent and partner TV viewing (0.59). It is possible that these home environment variables would be over-represented in the composite scores; therefore the results were checked by excluding these variables from the composites.

**Table 1:** Descriptive statistics for the home environment variables; means (SDs) and % (n) who responded yes.

Home food environment	N	
***Availability***		
Number of fruit types, mean (SD)^1^	1096	7.76 (3.20)
Number of vegetable types, mean (SD)^1^	1096	10.78 (3.74)
Number of energy-dense snack types, mean (SD)	1096	5.22 (2.08)
Presence of sugar-sweetened drinks, % (n)	1096	38.6 (423)
***Accessibility (visibility)*, *% (n)***		
Fruit on display^1^	1096	93.5 (1025)
Vegetables ready-to-eat^1^	1096	54.0 (592)
Energy-dense snacks on display	1096	20.5 (225)
Sugar-sweetened drinks on display	1096	6.6 (72)
***Accessibility (child can help him/herself)*, *% (n)***		
Fruit^1^	1096	53.4 (585)
Vegetables^1^	1096	28.3 (310)
Energy-dense snacks	1096	8.7 (95)
Sugar-sweetened drinks	1096	2.0 (22)
***Parental feeding practices*, *mean (SD)***		
Emotional feeding (1 = never; 5 = always)	1057	1.80 (0.62)
Instrumental feeding (1 = never; 5 = always)	1057	2.18 (0.66)
Encouragement^1^ (1 = never; 5 = always)	1057	4.12 (0.54)
Modelling^1^ (1 = never; 5 = always)	1057	3.63 (0.75)
Monitoring^1^ (1 = never; 5 = always)	1056	3.68 (0.91)
Covert restriction^1^ (1 = never; 5 = always)	1056	3.02 (0.83)
Restriction^1^ (1 = not at all; 7 = strictly)	1054	5.19 (1.11)
Family meal frequency (days per week)	1096	3.83 (1.62)
Frequency child eats while watching TV (days per week)	1096	1.32 (1.52)
**Home activity environment**		
Garden/outdoor space^1^, % (n)	1096	98.6 (1081)
Garden play equipment^1^, % (n)	1081	86.7 (950)
Allowed to play indoors^1^ (0 = never; 5 = all of the time), mean (SD)	1096	4.74 (0.61)
Allowed to play outdoors^1^ (0 = never; 5 = all of the time), mean (SD)	1081	4.35 (0.78)
Parental modelling of PA^1^, mean (SD)	1096	3.94 (0.76)
Parental support of PA^1^, mean (SD)	1096	3.99 (0.57)
**Home media environment**		
Number of media equipment, mean (SD)	1096	5.94 (2.90)
TV in the child’s bedroom, % (n)	1096	11.8 (129)
Household rules around media use^1^, % (n)	1096	66.1 (725)
TV viewing of respondent (hrs. per week), mean (SD)	1096	16.19 (9.37)
TV viewing of partner (hrs. per week), mean (SD)	1023	16.29 (8.82)

PA = physical activity.

^1^ Variable was identified as being associated with decreased risk for weight gain.

Constructs identified as being associated with decreased risk for weight gain were reverse-scored so that a higher total score on each composite would reflect higher risk for weight gain. Each variable was then standardized using z-scores. Missing values were recoded to 0 (the mean value for a standardized variable). There were few missing cases on home environment variables: 15 (1.4% of the total sample) for garden play equipment; 39 (3.6%) for emotional feeding, instrumental feeding, encouragement, and modelling of healthy eating; 40 (3.6%) for monitoring and covert restriction; and 42 (3.8%) for restriction. The only variable with more than 5% missing was partner TV viewing (these cases did not have a partner), and data were missing in just 73 cases (6.7%). There is concern that statistical analysis is likely to be biased when more than 10% of data are missing[26], but none of the home environment variables had this extent of missing data. The missing cases were assigned the mean score because this approach has been shown to provide a more accurate estimate of association than other methods of handling missing data[27]. The results were also checked by only including families with complete data. The standardized variables (z-scores) were then summed to create three composites: one for the home food environment (the sum of 21 food environment variables), one for the home activity environment (the sum of 6 activity environment variables), and one for the home media environment (the sum of 5 media environment variables). The food, activity, and media composites were then summed to create an overall home environment composite, dividing by the number of variables per composite so that each domain contributed equally to the overall score (food composite/21 + activity composite/6 + media composite/5).

Test-retest reliability (intraclass correlation coefficient (ICC); 95% confidence interval (CI)) of the home environment composites were acceptable to high: food (0.71; 0.52–0.83), activity (0.83; 0.72–0.91), media (0.92; 0.85–0.95), overall (0.92; 0.86–0.96). For ease of interpretation, each home environment composite was categorized into tertiles (three equal groups) for analysis, creating lower-, medium-, and higher-risk environment groups.

### Diet, physical activity, and TV viewing

Parents rated, on average, how often their child consumed fruit (excluding fruit juice), vegetables (excluding potatoes), energy-dense snacks (e.g. crisps and chocolate), sugar-sweetened drinks, artificially-sweetened drinks, fruit juice, and milk. All were recorded on an 8 point scale (1 = never or less than once a month; 8 = four or more times a day). The questions were based on those used in previous studies assessing preschool children’s food intake[28]. Activity level was assessed using the item: ‘compared to other children of the same age and sex, how physically active is your child?’ with a five-point response scale (1 = much less active; 5 = much more active); which has shown temporal stability from age 4 to 11, and correlated with objectively measured physical activity at age 11[29]. TV viewing was assessed using questions on duration of weekend and weekday TV viewing, which have previously correlated well with videotaped observations in 5-year-olds[19]. Responses were recorded in hours and minutes and averaged to create weekly TV viewing hours. Test-retest reliability was acceptable to high for all of the behaviors. The food and physical activity variables all had kappa values > 0.6 or percent agreement ≥ 60%, and for TV viewing, ICC was 0.87 (95% CI = 0.78–0.93).

Because the distributions of the diet, physical activity, and TV viewing variables were skewed, they were dichotomized for analysis. The diet and TV viewing variables were dichotomized using existing guidelines for preschool children. Fruit and vegetable consumption were each categorized so that the higher consumption group represented twice or more a day[30]. As child nutrition guidelines recommend that energy-dense snacks, specifically those high in fat, salt, or sugar, be consumed only very occasionally[31], the higher consumption group was defined as at least once a day. Similarly, as it is recommended that sugar- or artificially- sweetened drinks should rarely, if ever, be given to preschoolers[31], the higher consumption group was consumption of at least once a day. Guidelines for fruit juice and milk consumption are generally framed in terms of the amount or quality to be given[32]. As this information was not available, these variables were categorized in the same way as the other drink variables: at least once a day for fruit juice; at least twice a day for milk. In accordance with the American Academy of Pediatrics guidelines[33], the higher TV viewing group represented two or more hours per day. Physical activity level was categorized so that the active group included those who were more active (responses 4 (somewhat more active) and 5 (much more active)) than other children of the same age and sex; the comparison group were less active (responses 1 (much less active) and 2 (somewhat less active)) or about average (response 3).

### Anthropometric measurements at 4 years

Electronic weighing scales and height charts had been sent to all Gemini families when the children were 2 years old to collect measurements at 3-month intervals. At the time of the HEI, parents were also asked to provide their child’s height and weight measurements. Age- and sex-adjusted BMI standard deviation scores (SDS) were calculated using British growth reference data[34].

### Covariates

The following factors were identified as covariates as it was hypothesized that they may relate to the outcome variables (diet, physical activity, and TV viewing, and BMI at 4 years): child birth weight (recorded by health professionals) and maternal BMI (calculated from self-reported height and weight at baseline); maternal education level (also reported at baseline), categorized as higher (university education), intermediate (vocational or advanced high-school education), or lower (no qualifications or basic high-school education); and the child’s sex and their age at the time of the HEI.

### Statistical analyses

Associations between the home environment composites were examined using Pearson’s correlations. Logistic regression was used to examine associations between the domain-specific home environment composites and corresponding diet, physical activity, and sedentary behaviors (as shown in **Fig 1**). In each model, the home environment tertile assignment (lower-, medium-, higher-risk), was the grouping factor and child diet, physical activity, or TV viewing was the outcome. The child’s sex, age at the time of the HEI, and maternal BMI and education were included as covariates.

Analysis of covariance was used to examine associations between the overall home environment composite in addition to the separate food, activity, and media composites, and child BMI. In each model, the home environment tertile assignment (lower-, medium-, higher-risk), was the grouping factor and child BMI SDS was the outcome. The child’s birth weight, sex, age at the time of BMI measurement, and age at the time of the HEI, and maternal education level were included as covariates. The assumptions of normality and homogeneity of variance were met for all models, and there were no significant interaction effects between any of the covariates and the grouping variable. A p-value < 0.05 was considered statistically significant. All analyses were performed using SPSS version 18.0.

We could not examine whether the home environment composites predicted risk for overweight or obesity because just 80 (8.7%) of the sample met criteria for overweight or obesity using the International Obesity Task Force (IOTF) cut-offs[35].

## Results

### Descriptive characteristics

The total sample for analysis comprised 1096 families. Four-year height and weight data were available for 915 families (the rest of the HEI sample did not provide 4-year measurements), but there were no significant differences between these families and the total HEI sample on any of the other study variables. Parent characteristics for the study samples are shown in **Table 2**. All were the main caregivers of the child, 96.6% were mothers and 3.4% were fathers. Compared with the total Gemini sample (n = 2402), parents in the study samples were slightly older (34 vs. 33 years), more had higher education (48.3 and 50.2% vs. 41.9%), more were white (95.2 and 95.3% vs. 92.9%), and they had a slightly lower BMI (24.8 and 24.7 vs. 25.1) at baseline.

**Table 2:** Descriptive characteristics for the study samples (% (n) unless stated otherwise).

Total sample	N = 1096
Maternal education level	
Low	15.4 (169)
Mid	36.3 (398)
High	48.3 (529)
Maternal BMI (mean (SD))	24.84 (4.58)
Child’s sex	
Male	50.1 (549)
Female	49.9 (547)
Child’s age, yrs. (mean (SD))	4.17 (0.40)
Child’s diet behaviors	
*Fruit consumption*	
≥ twice a day	77.7 (852)
< twice a day	22.3 (244)
*Vegetable consumption*	
≥ twice a day	51.2 (561)
< twice a day	48.8 (535)
*Energy-dense snack consumption*	
≥ once a day	13.4 (147)
< once a day	86.6 (949)
*Sugar-sweetened drink consumption*	
≥ once a day	10.7 (117)
< once a day	89.3 (979)
*Artificially-sweetened drink consumption*	
≥ once a day	52.5 (575)
< once a day	47.5 (521)
*Fruit juice consumption*	
≥ once a day	48.4 (531)
< once a day	51.6 (565)
*Milk consumption*	
≥ twice a day	64.1 (702)
< twice a day	35.9 (394)
*Physical activity level* ^*1*^	
Somewhat or much more active	61.0 (669)
About average or less active	39.0 (427)
*TV viewing*	
≥ 2 hours per day	39.1 (428)
< 2 hours per day	60.9 (668)
**Sample with 4-year BMI**	**N = 915**
Maternal education level	
Low	14.3 (131)
Mid	35.5 (325)
High	50.2 (459)
Maternal BMI (mean (SD))	24.74 (4.51)
Child’s sex	
Male	49.9 (457)
Female	50.1 (458)
Child’s age, yrs. (mean (SD))	4.14 (0.40)
Birth weight, kg (mean (SD))	2.47 (0.53)
4-year BMI SDS (mean (SD))	-0.06 (0.97)

BMI = body mass index; SDS = standard deviation score.

^1^Compared to other children of the same age and sex.

Child characteristics for the study samples are also shown in **Table 2**. Children were on average 4 years old at the time of the HEI. Approximately three-quarters (78%) consumed fruit at least twice a day, and approximately half (51%) consumed vegetables this frequently. A minority (10%) of children consumed energy-dense snacks at least once a day. Almost two thirds (61%) of children were reported to be more physically active than other children of the same age and sex. More than a third (39%) of children watched two or more hours of TV a day.

The ranges (for the standardized scores) on each home environment composite indicated that there was considerable variation: food (-19.25–25.25), activity (-4.93–16.58), media (-7.19–18.11), overall (-2.44–4.01). Associations between the composites were moderate or low: r = 0.27 for food and activity (p < 0.001), 0.30 for food and media (p < 0.001), and 0.05 for activity and media (ns).

### Associations between the home environment and diet, physical activity, and TV viewing

As shown in **Table 3**, children living in higher-risk home food environments were less likely to consume fruit and vegetables, and more likely to consume energy-dense snacks and sugar-sweetened drinks than those living in lower-risk home food environments (all p’s < 0.001). There were no significant associations between the home food environment and artificially-sweetened drink, fruit juice, or milk consumption.

**Table 3:** Associations between the home food, activity, and media environment tertiles and corresponding diet, physical activity, and sedentary behaviors (N = 1096)^1^.

	Lower-risk		Mid-risk			Higher-risk		
	environment		environment			environment		
	%	OR	%	OR	P-value	%	OR	P-value
	(n)		(n)	(95% CI)		(n)	(95% CI)	
**Dietary behaviors**								
Fruit (≥ twice a day)	85.5	1.00	79.2	0.67	0.041	68.6	0.39	<0.001
	(312)		(289)	(0.45–0.98)		(251)	(0.27–0.57)	
Vegetables (≥ twice a day)	61.9	1.00	51.0	0.66	0.008	40.7	0.47	<0.001
	(226)		(186)	(0.49–0.90)		(149)	(0.34–0.64)	
Energy-dense snacks (≥ once a day)	7.1	1.00	11.2	1.63	0.064	21.9	3.48	<0.001
	(26)		(41)	(0.97–2.75)		(80)	(2.16–5.62)	
Sugar-sweetened drinks (≥ once a day)	6.3	1.00	7.9	1.33	0.334	17.8	3.49	<0.001
	(23)		(29)	(0.75–2.34)		(65)	(2.10–5.81)	
Artificially-sweetened drinks (≥ once a day)	50.4	1.00	52.3	1.00	0.989	54.6	0.96	0.809
	(184)		(191)	(0.74–1.36)		(200)	(0.71–1.31)	
Fruit juice (≥ once a day)	49.0	1.00	49.9	1.05	0.751	46.4	0.95	0.728
	(179)		(182)	(0.78–1.40)		(170)	(0.71–1.28)	
Milk (≥ twice a day)	94.5	1.00	94.5	1.05	0.872	93.4	0.80	0.492
	(345)		(345)	(0.55–2.01)		(342)	(0.43–1.50)	
**Activity behaviors**								
Physical activity (more active)	70.7	1.00	61.0	0.64	0.004	51.4	0.43	<0.001
	(258)		(224)	(0.47–0.87)		(187)	(0.32–0.59)	
**Media behaviors**								
TV viewing (≥ 2 hrs. per day)	21.8	1.00	35.1	1.71	0.002	60.1	3.51	<0.001
	(79)		(128)	(1.21–2.40)		(221)	(2.48–4.96)	

^1^Adjusting for maternal education level, maternal BMI, the child’s age at the time of the HEI, and the child’s sex; OR = odds ratio; 95% CI = 95% confidence interval; 1.00 denotes the reference group.

Children living in higher-risk home activity environments were less active than children living in lower-risk home activity environments (p-value < 0.001). Children living in higher-risk home media environments were more likely to watch TV for at least two hours a day than children living in lower-risk home media environments (p-value < 0.001).

In all cases where there was a significant difference between the two extreme groups (higher- and lower-risk home environments), the OR for the mid-risk group was in between. All the results were the same when including only the child’s sex and age as covariates, when individually excluding those variables with an inter-correlation of (≥0.50), and when only including families with data on all of the home environment variables.

### Associations between the home environment and BMI SDS

As shown in **Table 4**, there was no significant association between the overall home environment composite and BMI SDS at 4 years. Associations between the separate composites and 4-year BMI SDS were also non-significant. The results were the same when including only the child’s sex and age as covariates, when individually excluding those variables with an inter-correlation of (≥0.50), and when only including families with data on all of the home environment variables.

**Table 4:** Associations between the home environment tertiles and BMI SDS at 4 years^1^ (N = 915).

	Adjusted mean (SD)	F_df_ (p-value)
**Overall environment**		
Lower-risk	-0.03 (1.78)	0.61_2,906_ (0.545)
Mid-risk	-0.10 (1.75)	
Higher-risk	-0.10 (1.72)	
**Food environment**		
Lower-risk	-0.01 (1.78)	2.01_2,906_ (0.135)
Mid-risk	-0.16 (1.72)	
Higher-risk	-0.05 (1.72)	
**Activity environment**		
Lower-risk	-0.11 (1.75)	0.48_2,906_ (0.619)
Mid-risk	-0.09 (1.72)	
Higher-risk	-0.03 (1.75)	
**Media environment**		
Lower-risk	-0.02 (1.81)	1.11_2,906_ (0.331)
Mid-risk	-0.06 (1.72)	
Higher-risk	-0.14 (1.78)	

^1^ Adjusting for the child’s age at the time of the BMI measurement, age at the time of the HEI, birth weight, and sex, and maternal education level.

## Discussion

This is the first study to examine associations between composite indicators of the home environment and both weight-relevant behaviors: diet, physical activity, and sedentary time, and BMI in a large sample of preschool children. There were clear associations between the environment composite measures and the related behaviors (diet, physical activity, and TV viewing), but none of the home environment composites were associated with BMI.

The home food environment composite correlated positively with the activity and media composites, although the correlations were moderate, suggesting that higher risk in one domain is to some extent reflected in others. The very small association between the home media and activity composites indicates that children may live in homes that present risk for weight gain in some respects but not others, highlighting the importance of using composite measures to capture a more comprehensive picture of the obesogenic home environment. This finding is also in line with the view that physical activity and sedentary behavior are separate constructs with separate influences[4].

Just three previous studies have examined associations between composite measures of the home environment and child or adolescent BMI[7,12,14], and only one of them used a comprehensive, ‘pure’ home environment measure[7]. This study found an association between the home environment and BMI in a sample of older children (mean age = 10 years, range = 5–17 years); although the analyses were not adjusted for covariates. The null associations in the present study do not discount the relevance of the home environment to weight trajectories, because influences on BMI may generally emerge later in development. Indeed, other research has found no associations with BMI in younger children (5–6 years), but some associations in older children (10–12 years)[36].

This may be because home environmental influences get stronger over time. Although younger children tend to spend more time in the home environment than older children and adolescents, the latter may have greater exposure to some obesogenic influences if parents are generally more restrictive with younger children. Certain psychosocial aspects of the home environment may have greater relevance for BMI in adolescence when there are important emotional transitions[37]. Older children and adolescents are also exposed to a wider range of obesogenic influences outside of the home, which may have a cumulative effect on BMI. For example, exposure to the school environment and new peer groups may add to the risk for weight gain. Future research should examine associations between composite indicators of the home environment and BMI over a longer time period, while taking into account external influences.

It is also possible that associations between the home environment and weight may only appear among those who are genetically susceptible to weight gain. Weight is known to have a strong genetic basis[38], and there is evidence that genetic risk influences responses to the environment[39]. However, no studies have directly tested genetic moderation within the context of the overall obesogenic home environment.

Although limited, some studies have found associations between aspects of the home environment and BMI in children as young as 4 years of age. For example, having a TV in the bedroom was associated with increased risk for overweight in a sample of 2761 American children[10] and not eating dinner at least 6 days per week was associated with increased risk for obesity in another sample of 8550 American children[11]. These study samples were more diverse (in terms of ethnic background and/or socioeconomic status) than the present study and may have been better powered to detect an association with weight. Research indicates that lower socioeconomic status groups and some ethnic minority groups may live in more obesogenic home environments[40,41] and may be heavier than other demographic groups[42,43]. In the study by Dennison and colleagues, almost half of the sample had a TV in their bedroom (n = 1380), compared to just 12% in the present study. In the study by Anderson and Whitaker, 1573 (18%) children were obese, while just 80 (9%) were classified as being overweight or obese in the present sample. Although the present study examined associations with composite home environment measures, which may be better powered to detect an effect than when focusing on individual aspects, the range of scores may still have been somewhat narrow. It is also noteworthy that the studies described above were carried out in the US, where the environment factors may be even stronger than in the UK. For example, although the proportion of adverts promoting ‘non-core’ foods is high in many countries, it is particularly high in the US[44].The associations between the home environment and diet, physical activity, and TV viewing support the notion that the home is an important setting for obesity prevention[5,6]. There is currently little evidence supporting the effectiveness of home-based child obesity interventions, but this may be because few studies have intervened on multiple levels of the home environment[45]. In adults, there is some evidence that weight-loss programs targeting multiple aspects of the home environment produce better weight-loss outcomes than standard behavioral programs; although, these weight-loss outcomes were not maintained[46]. It seems important to clarify the role of the home environment in weight trajectories so that home-based interventions can be appropriately delivered. Nevertheless, modifying the home environment to target diet, physical activity, and sedentary time are important in their own right as well as possibly being a first step towards obesity prevention.

There are several possible pathways by which the home environment might influence BMI. This study focused on associations between directly corresponding home environment domains and behaviors. However, each domain might (directly or indirectly) influence several weight-related behaviors, which in turn influence BMI. In line with this, aspects of the media environment have been associated with child diet[47], physical activity[48], and sleep[49] in addition to TV viewing, suggesting several potential explanatory mechanisms. Weight-related behaviors are also inter-related. For example, TV viewing itself may act as a trigger for eating. This association may be established from a young age if, for example, parents place their child in front of the TV with a snack or meal while they do household chores[50]. Children may also associate TV viewing with eating because the content of TV shows or adverts may trigger snacking. Exposure to TV advertisements influences the type of food desired, requested and consumed[51], and the branding used in these adverts can have powerful effects on eating behaviors[52].

## Strengths and Limitations

The strengths of this study include a large sample, a focus on the preschool period, the ability to control for various potential confounding factors, a comprehensive measure of the home environment, and composite indicators of the home environment guided by feedback from an expert panel. The construction of composite scores is a complicated exercise, which involves several stages where subjective judgement has to be made: selection of variables to be included, treatment of missing values, choice of aggregation method, and choice of weights to apply to each variable[53]. In the present study, although the home environment composites were comprehensive in that they incorporated many factors agreed to be relevant to risk for weight gain, it is possible that some relevant factors were not included. The z-score standardization procedure was selected as the most appropriate aggregation method as it standardizes all variables while retaining a greater level of information than other methods would. However, this approach is typically applied to continuous and ordinal variables, and the z-scores of dichotomous variables depend on the proportion of 0s and 1s in the data. It is possible that extreme z-scores had an undue influence on individuals' composite scores. Assigning differential weights based on the ‘desirability’ of scores on each variable is one way to correct for this; however, there is currently insufficient information from the literature to determine differential weighting for the home environment variables. In any case, existing research has shown that unweighted composites are highly correlated with, and perform as well as weighted composites, especially when the number of variables is large[54]. Moreover, the results were the same when using different versions of the composite scores, providing some support for their robustness. Nevertheless, it would be useful to further examine how differently-constructed composites are related and how they each perform in classifying higher- versus lower-risk home environments.As with many other studies in this area, the study used parent-report measures, and the home environment and diet, physical activity, and TV viewing were assessed concurrently, which may have introduced some bias. However, the measures demonstrated moderate to high test-retest reliability, and the findings concur with those where the home environment and diet, physical activity, and TV viewing were assessed on separate occasions[55]. The measures assessing child physical activity and TV viewing, and the physical aspects of the home environment have been validated previously[16,19,29], but the questions on parental policies and child diet have not been validated. As the associations in this study were cross-sectional, causal inferences cannot be made. Children’s own preferences could influence the environment to some extent. Although height and weight measurements are preferably taken by health professionals, this was not feasible in the present study given the sample size. However, parents can provide accurate measurements if they measure the child themselves at home[56], as was done in the present study by providing scales and height charts. BMI is an important indicator of weight status, but other measures of body fat such as skinfold thickness and waist circumference can provide further information on body composition[57].

## Conclusions

This study found clear associations between composite measures of the home environment and diet, physical activity, and TV viewing in preschool children, but there were no significant associations with BMI. Future research should examine whether home environment influences on BMI only emerge as children get older, and whether they are restricted to genetically susceptible individuals.

## Supporting Information

S1 File (DOCX)
