# Supplementary material for: The Obesogenic Quality of the Home Environment: Associations with Diet, Physical Activity, TV Viewing, and BMI in Preschool Children
Source: PLoS One. 2015 Aug 6;10(8):e0134490. doi: 10.1371/journal.pone.0134490 (PMC4527827; doi:10.1371/journal.pone.0134490)
Supplement: S1 File — (DOCX) [file pone.0134490.s001.docx]

**Home Environment Interview (HEI)**

Highlighted text is text interviewer needs to read out loud, other text is for coding purposes and may not need to be read out loud.

**Section A - GENERAL INFORMATION QUESTIONS**

Today’s date: __ / __ / __

Family ID Number:

**A1.** Please can I speak to <Named Contact>?

If first phone call:

Hello, this is <researcher name> calling on behalf of the Gemini twin study. Instead of a questionnaire, we are carrying out this part of the study over the phone. Is now a good time to talk?

If not convenient, arrange another time that is convenient and record this in the call attempts excel spreadsheet. If the participant doesn’t want to do the interview, also record this in the call attempts spreadsheet.

If yes, proceed as below.

We have the twins’ names registered as <twin1 name> and <twin2 name>, is that correct and what you would usually call them? Is <twin1 name> the first born twin and <twin2 name> the second born?

If yes to names and birth order: click ‘NEXT’ button.

If no to names or birth order: check Gemini ID and insert correct names (in the correct

order):

twin 1: ……… twin 2 ……………..

I would like to ask you some questions about <twin1 name> and <twin2 name> and your home. Ideally we need to talk to the person who is responsible for the majority of the food shopping and childcare within the home. Do you think you will be in a position to answer these questions?

If FOLLOW-UP phone call:

Hello, this is <researcher name> calling on behalf of the Gemini twin study. We spoke recently and you agreed to take part in a telephone interview. Is now a good time to talk?

1. If OK to talk and speaking to <named contact>: click ‘NEXT button’

2. If OK to talk and NOT speaking to <named contact> fill in name below and click ‘NEXT’ button.

Could I take your name?

First Name ……………….. Last Name ………………...

3. If NOT OK to talk, arrange a convenient time to call back, make a note of this time and click ‘BACK’.

Thank you for taking the time to talk, the interview should take around 30 minutes to complete. Just to give you some background, the aim of the interview is to get a picture of the environment young children are growing up in. There are no right or wrong answers so please just answer honestly. If there are any questions you need me to clarify, or any other information you think would be relevant then please feel free to stop me at any time. All your responses will be kept confidential and anonymous.

**A2.** Please could you confirm the twin’s date of birth?

Insert correct date of birth ____ / ____ / _____

**A3.** Please could you confirm your relationship with <twin1 name> and <twin2 name>?

□ Mother

□ Father

□ Guardian

□ Same sex partner

□ Grandparent

□ Nanny

□ Other, please specify: ……………………………………………………

**A4.** Please could you confirm your home address? Insert correct address.

Address : ……………………………

……………………………

……………………………………

……………………………………

Postcode : ………………………………

**A5.** How many adults, including yourself, currently live in your home? Only include people who are aged 18 years or older and who live in your home all of the time.

………….. Adults

**A6.** Does this include…

Your husband? Yes □ No □

Your wife? Yes □ No □

Your partner? Yes □ No □

For female participants ask: Does this include your husband? If yes, select no for wife and partner. If no, then ask ‘your partner?’

For male participants ask: Does this include your wife? If yes, select no for husband and partner. If no, then ask ‘your partner?’

**A7.** How many children, under 18 years of age, not including <twin1 name> and <twin2 name> currently live in your home?

………….. Children

*If no other children, skip A8.*

**A8.** Since the birth of <twin1 name> and <twin2 name>, have any additional children joined the household? *If no, skip A9.*

Yes □ No □

**A9.** Please can you give the name, date of birth and sex of each additional child?

Complete the table below accordingly.

|  | Child’s name | Date of Birth | Sex |
| --- | --- | --- | --- |
| 1 |  |  | male □ female □ |
| 2 |  |  | male □ female □ |
| 3 |  |  | male □ female □ |
| 4 |  |  | male □ female □ |

| Additional comments about changes to family circumstances. |
| --- |
|  |

**Section B – HOUSE AND NEIGHBOURHOOD**

The next few questions are about where you live.

**B1.** Which of the following options best describes the type of home you live in?

Read out each of the options below.

□ Flat (which floor……)

□ Semi-detached house

□ Terraced house

□ Detached house

□ Or other, please describe: …………..…………………………………

**B2.** Do you have stairs in your home?

Yes □ No □

**B3.** Would you say that your home is on a busy street with lots of traffic?

Yes □ No □

Any comments on this section (B1-B3): ………………………………

I’m now going to ask some questions about how satisfied you are with where you live.

For each question please choose a score from 1 to 5. A score of 1 means strongly dissatisfied, 2 means somewhat dissatisfied, 3 means neither satisfied nor dissatisfied, 4 means somewhat satisfied and 5 means strongly satisfied.

To make sure the participant is ranking correctly, for the first question of each set of questions where there are a number of response options, repeat their response back to them e.g. if participant says 5, interviewer says ‘so that’s 5, strongly satisfied?’ etc. If the participant asks what we mean by neighbourhood, say that it is what they perceive their neighbourhood/local area to be.

**B4.** How satisfied are you with the quality of schools in your neighbourhood? This includes preschool and nursery.

If the participant says that they are strongly satisfied with the preschools in their neighbourhood but strongly dissatisfied with the secondary schools in their neighbourhood, get them to consider this with their response. For example, they may give a neutral score of 3 when taking this into account.

1 2 3 4 5

**B5.** How satisfied are you with access to entertainment in your neighbourhood such as restaurants and cinemas?

If participants say there aren't any restaurants/cinemas in their neighbourhood, ask them how satisfied they are with this.

1 2 3 4 5

**B6.** How satisfied are you with the safety of your neighbourhood? By this we mean safety from threat of crime.

1 2 3 4 5

**B7.** How satisfied are you with the level of traffic in your neighbourhood?

1 2 3 4 5

**B8.1** How satisfied are you with the number of food shops in your neighbourhood?

1 2 3 4 5

**B8.2** How satisfied are you with the quality of food shops in your neighbourhood?

1 2 3 4 5

**B9.1** How satisfied are you with the number of restaurants in your neighbourhood?

This includes all types of restaurants, sit-in or take-away.

If participants say there aren't any restaurants in their neighbourhood, ask them how satisfied they are with this.

1 2 3 4 5

**B9.2** How satisfied are you with the quality of restaurants in your neighbourhood?

Again this includes all types of restaurants, sit-in or take-away.

1 2 3 4 5

**B10.** How satisfied are you with your neighbourhood as a place to raise children?

1 2 3 4 5

**B11.** How satisfied are you with your neighbourhood as a place to live?

1 2 3 4 5

**B12.** How easy it is to walk in your neighbourhood, with 1 being not at all easy and 5 being very easy?

1 2 3 4 5

**B13.** How easy it is to bicycle in your neighbourhood, with 1 being not at all easy and 5 being very easy?

1 2 3 4 5

**Section C – PHYSICAL ACTIVITY ENVIRONMENT**

The next section is about activity facilities available to you.

**C1.** Are there any parks or outdoor recreation areas close to your home?

If the participant asks what we mean by ‘close’ say that we mean parks or outdoor recreation areas that they believe are within a reasonable walking distance from their home or a short drive away.

Yes □ No □ Don’t know □

**C2.** Are there any in-door recreation centres, for example a gym or indoor soft play close to your home?

If the participant asks what we mean by ‘close’ say that we mean indoor recreation centres that they believe are within a reasonable walking distance from their home or a short drive away.

Yes □ No □ Don’t know □

**C3.** Do you have a garden or outdoor space that <twin1 name> and <twin2 name> can play in?

This includes shared garden space for people living in flats, but does not include park space, even if it is very close to home.

Yes □ No □

*If no skip C4, C5.*

**C4.** Would you say that your garden or (outdoor space) is small, medium or large?

This is a subjective question. The participant should say what they feel the size of their garden is.

small □ medium □ large □

**C5.** Do you have any usable play equipment such as swings, slides, climbing frames, trampolines in your garden or (outdoor space)?

This includes sandpits. Usable means that it is ready to use. For example, swings are well grounded and have chairs.

Yes □ No □

**C6.** Do <twin1 name> and <twin2 name> each have a usable tricycle, bike, scooter or wheeled toy?

Usable means that it is ready to use. For example, bikes have tires that are pumped up and chains that are not broken.

Yes (both) □ No □ Yes <twin1 name> □ Yes <twin2 name> □

For the next two questions, again please choose a score from 1 to 5: 1 means never, 2 means rarely, 3 means some of the time, 4 means most of the time, 5 means all of the time.

**C7.** How often would you say that <twin1 name> and <twin2 name> are allowed to play actively in your garden or outdoor space?

For the first question, to make sure the participant is ranking correctly, repeat their response back to them e.g. if participant says 5, interviewer says ‘so that’s 5, all of the time?’ etc.

A potential response may be that the child is only allowed to play outside if an adult is present. If play is never restricted within that parameter, tick 5 all of the time.

Explanations for C7 and C8 are irrelevant. It might be that participants rarely allow play in the garden because they do not feel that it is safe. This response should remain as 2, rarely.

1 2 3 4 5

**C8.** How often would you say that <twin1 name> and <twin2 name> are allowed to play actively inside the home?

1 2 3 4 5

**C9.** Compared to other children of the same age and sex, how physically active are <twin1 name> and <twin2 name>? Please choose a score from 1 to 5: 1 means much less active, 2 means somewhat less active, 3 means about average, 4 means somewhat more active, 5 means much more active.

<twin1 name>: 1 2 3 4 5

<twin2 name>: 1 2 3 4 5

**Section D – PARENTAL MODELLING OF ACTIVITY**

For the next section, again please choose a score from 1 to 5. 1 means never, 2 means rarely, 3 means sometimes, 4 means often, 5 means very often. For each question, please indicate whether your response is the same or different for <twin1 name> and <twin2 name>.

Throughout this section, physical activity means any kind of physical activity including moderate e.g. walking and vigorous e.g. running.

**D1.** How often do you or your <husband/wife/partner> encourage <twin1 name> and <twin2 name> to do physical activity?

For the first question, to make sure the participant is ranking correctly, repeat their response back to them e.g. if participant says 5, interviewer says ‘so that’s 5, very often?’ etc. If parents say 1 because they don’t need to as their child is already physically active, still keep response as 1. In other words, it doesn’t matter what the reason is.

1 2 3 4 5

If different arrangement for twins: <twin2 name>: 1 2 3 4 5

If the participant does not indicate whether their response is the same or different for <twin1 name> and <twin2 name>, prompt them to check.

**D2.** How often do you or your <husband/wife/partner> do physical activity or play sports with <twin1 name> and <twin2 name>?

1 2 3 4 5

If different arrangement for twins: <twin2 name>: 1 2 3 4 5

If the participant does not indicate whether their response is the same or different for <twin1 name> and <twin2 name>, prompt them to check.

**D3.** How often do you or your <husband/wife/partner> provide transport to a place where <twin1 name> and <twin2 name> can do physical activity?

1 2 3 4 5

If different arrangement for twins: <twin2 name>: 1 2 3 4 5

If the participant does not indicate whether their response is the same or different for <twin1 name> and <twin2 name>, prompt them to check.

**D4.** How often do you or your <husband/wife/partner> watch <twin1 name> and <twin2 name> participate in physical activity?

1 2 3 4 5

If different arrangement for twins: <twin2 name>: 1 2 3 4 5

If the participant does not indicate whether their response is the same or different for <twin1 name> and <twin2 name>, prompt them to check.

**D5.** How often do you or your <husband/wife/partner> tell <twin1 name> and <twin2 name> that being physically active is good for their health?

1 2 3 4 5

If different arrangement for twins: <twin2 name>: 1 2 3 4 5

If the participant does not indicate whether their response is the same or different for <twin1 name> and <twin2 name>, prompt them to check.

**D6.** How often do you or your <husband/wife/partner> try to be active in front of <twin1 name> and <twin2 name>?

This includes occasions where the child sees their parent(s) preparing to exercise, even if they are not able to actually see them exercise.

1 2 3 4 5

If different arrangement for twins: <twin2 name>: 1 2 3 4 5

If the participant does not indicate whether their response is the same or different for <twin1 name> and <twin2 name>, prompt them to check.

**D7.** How often do you or your <husband/wife/partner> try to show enthusiasm about being active?

1 2 3 4 5

If different arrangement for twins: <twin2 name>: 1 2 3 4 5

If the participant does not indicate whether their response is the same or different for <twin1 name> and <twin2 name>, prompt them to check.

**D8.** How often do you or your <husband/wife/partner> show <twin1 name> and <twin2 name> how much you enjoy being active?

1 2 3 4 5

If different arrangement for twins: <twin2 name>: 1 2 3 4 5

If the participant does not indicate whether their response is the same or different for <twin1 name> and <twin2 name>, prompt them to check.

**Section E - MEDIA**

The next section is about the media equipment you have in your home

**E1.** How many working TV’s do you have in your home?

Include TV’s that are temporarily broken if there is a plan to get them fixed.

…….. (enter 99 if Don’t know, enter 0 if none) *If 0, skip E2 and E5*

**E2.** Do you have cable or satellite?

This does not include freeview.

Yes □ No □

**E3.** How many working VCR or DVD players do you have in your home?

Include VCR’s or DVD players that are temporarily broken if there is a plan to get them fixed. Also include DVD players within computers if they are used to watch films on.

…... (enter 99 if Don’t know, enter 0 if none, if 0 to E1 and 0 to E3, skip E4)

**E4.1** On average, how long do <twin1 name> and <twin2 name> watch TV or DVDs during the following times of a typical weekday (Monday to Friday), at this time of year?

Only include TV viewing in the home. Write hours and minutes. If less than one hour e.g. 15 minutes put 0 hours and 15 minutes. If 1 hour, put 1 hour and 0 minutes. For E4.1 – E4.6, read out each of the times (e.g. morning (6am to 12 noon)) in turn and wait for the participant’s response before reading out the next time.

Morning (6am to 12 noon) ……. hours ……. minutes per day

Afternoon (12am to 6pm) …….. hours ……. minutes per day

Evening (6pm to midnight) ……. hours ……. minutes per day

If different arrangement for twins, enter answers for <twin1 name> above and for <twin2 name> below:

Morning (6am to 12 noon) ……. hours ……. minutes per day

Afternoon (12am to 6pm) …….. hours ……. minutes per day

Evening (6pm to midnight) ……. hours ……. minutes per day

If the participant does not indicate whether their response is the same or different for <twin1 name> and <twin2 name>, prompt them to check.

**E4.2** On average, how long do <twin1 name> and <twin2 name> watch TV or DVDs during the following times of a typical weekend day, at this time of year?

Only include TV viewing in the home. Write hours and minutes. If less than one hour e.g. 15 minutes put 0 hours and 15 minutes. If 1 hour, put 1 hour and 0 minutes.

Morning (6am to 12 noon) ……. hours ……. minutes per day

Afternoon (12am to 6pm) …….. hours ……. minutes per day

Evening (6pm to midnight) ……. hours ……. minutes per day

If different arrangement for twins, enter answers for <twin1 name> above and for <twin2 name> below:

Morning (6am to 12 noon) ……. hours ……. minutes per day

Afternoon (12am to 6pm) …….. hours ……. minutes per day

Evening (6pm to midnight) ……. hours ……. minutes per day

If the participant does not indicate whether their response is the same or different for <twin1 name> and <twin2 name>, prompt them to check.

**E4.3** On average, how long do you watch TV or DVDs during the following times of a typical weekday (Monday to Friday), at this time of year?

Only include TV viewing in the home. Write hours and minutes. If less than one hour e.g. 15 minutes put 0 hours and 15 minutes. If 1 hour, put 1 hour and 0 minutes.

Morning (6am to 12 noon) ……. hours ……. minutes per day

Afternoon (12am to 6pm) …….. hours ……. minutes per day

Evening (6pm to midnight) ……. hours ……. minutes per day

**E4.4** On average, how long do you watch TV or DVDs during the following times of a typical weekend day, at this time of year?

Only include TV viewing in the home. Write hours and minutes. If less than one hour e.g. 15 minutes put 0 hours and 15 minutes. If 1 hour, put 1 hour and 0 minutes.

Morning (6am to 12 noon) ……. hours ……. minutes per day

Afternoon (12am to 6pm) …….. hours ……. minutes per day

Evening (6pm to midnight) ……. hours ……. minutes per day

**E4.5** On average, how long does your <husband/wife/partner> watch TV or DVDs during the following times of a typical weekday (Monday to Friday), at this time of year? Only include TV viewing in the home. Write hours and minutes. If less than one hour e.g. 15 minutes put 0 hours and 15 minutes. If 1 hour, put 1 hour and 0 minutes.

Morning (6am to 12 noon) ……. hours ……. minutes per day

Afternoon (12am to 6pm) …….. hours ……. minutes per day

Evening (6pm to midnight) ……. hours ……. minutes per day

**E4.6** On average, how long does your <husband/wife/partner> watch TV or DVDs during the following times of a typical weekend day, at this time of year?

Only include TV viewing in the home. Write hours and minutes. If less than one hour e.g. 15 minutes put 0 hours and 15 minutes. If 1 hour, put 1 hour and 0 minutes.

Morning (6am to 12 noon) ……. hours ……. minutes per day

Afternoon (12am to 6pm) …….. hours ……. minutes per day

Evening (6pm to midnight) ……. hours ……. minutes per day

**E5.** Do <twin1 name> and <twin2 name> have a working TV in their bedroom?

Include TV’s if it is a shared bedroom and the TV belongs to another child.

Yes □ No □

If different arrangement for twins: <twin2 name>: Yes □ No □

If the participant does not indicate whether their response is the same or different for <twin1 name> and <twin2 name>, prompt them to check.

**E6.** How many working computers or laptops do you have in your home?

Include computers or laptops that are temporarily broken if there is a plan to get them fixed.

…….. (enter 0 if none) *If 0, skip E7*

**E7.** Do <twin1 name> and <twin2 name> have a computer or laptop in his/her bedroom? Include computers if it is a shared bedroom and the computer belongs to another child.

Yes □ No □

If different arrangement for twins: <twin2 name>: Yes □ No □

If the participant does not indicate whether their response is the same or different for <twin1 name> and <twin2 name>, prompt them to check.

**E8.** How many working games consoles, such as Play Station, Nintendo DS, Wii do you have in your home?

Include game consoles that are temporarily broken if there is a plan to get them fixed. This includes hand held games consoles.

…….. (enter 0 if none) *If 0, skip E9, if 0 to E1, E6, and E8, skip E9-E13*

**E9.** Do <twin1 name> and <twin2 name> have a games console in their bedroom? Include games consoles if it is a shared bedroom and the games console belongs to another child.

Yes □ No □

If different arrangement for twins: <twin2 name>: Yes □ No □

If the participant does not indicate whether their response is the same or different for <twin1 name> and <twin2 name>, prompt them to check.

**E10.** Do you have any rules around TV watching or computer use for <twin1 name> and <twin2 name>?

Yes □ No □

**E11.** Do you ever reward good behaviour with extra TV or computer time?

Yes □ No □

If different arrangement for twins: <twin2 name>: Yes □ No □

If the participant does not indicate whether their response is the same or different for <twin1 name> and <twin2 name>, prompt them to check.

**E12.** Do you ever reduce TV or computer time if <twin1 name> or <twin2 name> is naughty?

Yes □ No □

If different arrangement for twins: <twin2 name>: Yes □ No □

If the participant does not indicate whether their response is the same or different for <twin1 name> and <twin2 name>, prompt them to check.

**E13.** Do <twin1 name> and <twin2 name> ever eat while watching TV? This includes meals and snacks that are eaten in front of the TV.

If the participant says sometimes, check whether this is on a weekly basis. If not on a weekly basis, enter no.

Yes □ No □ *If No skip E14-E17*

If different arrangement for twins: <twin2 name>: Yes □ No □

If the participant does not indicate whether their response is the same or different for <twin1 name> and <twin2 name>, prompt them to check.

**E14.** How many days per week do <twin1 name> and <twin2 name> eat breakfast while watching TV?

0 1 2 3 4 5 6 7

If different arrangement for twins: <twin2 name>: 0 1 2 3 4 5 6 7

**E15.** How many days per week do <twin1 name> and <twin2 name> eat a midday meal while watching TV?

0 1 2 3 4 5 6 7

If different arrangement for twins: <twin2 name>: 0 1 2 3 4 5 6 7

**E16.** How many days per week do <twin1 name> and <twin2 name> eat an evening meal while watching TV?

0 1 2 3 4 5 6 7

If different arrangement for twins: <twin2 name>: 0 1 2 3 4 5 6 7

**E17.** How many days per week do <twin1 name> and <twin2 name> eat snacks while watching TV?

0 1 2 3 4 5 6 7

If different arrangement for twins: <twin2 name>: 0 1 2 3 4 5 6 7

**Sections F - L: FOOD AVAILABLILTY**

The next section is about food and drink that is currently in your home. For the food and drink that we ask about, please include all items that are in your home even if **<twin1 name> and <twin2 name>** don’t eat or drink them. If you are unsure of any of the answers, please have a look to see what is in your home. If you have a phone in the kitchen and would like to move there now, that may help. Please answer as accurately as possible.

**Fruit**

**F.1.1.** Do you have any fresh fruit in your home now?

Yes □ No □

**F.1.2.** If yes, what types of fresh fruit do you have in your home now?

This is an open question. As the participant lists the fresh fruit they have, tick the matching options in the table or add any other fresh fruit to the free entry box which says other.

When the participant finishes, prompt her/him by reminding her/him of places she/he may have forgotten: Have you remembered fresh fruit in your fridge, in a fruit bowl and in your cupboards?

| Fresh fruit |
| --- |
| List of standard fruits to choose from (see below) as well as a free-entry box for less common items.   \|  \| Yes/No \| \| --- \| --- \| \| Apples \|  \| \| Bananas \|  \| \| Cherries \|  \| \| Grapefruit \|  \| \| Grapes \|  \| \| Kiwi \|  \| \| Mangoes \|  \| \| Melon \|  \| \| Nectarines \|  \| \| Oranges/tangerines/clementines/mandarins \|  \| \| Peaches \|  \| \| Pears \|  \| \| Pineapple \|  \| \| Plums \|  \| \| Strawberries \|  \|  \| Other fresh fruit \| Number of other items \| \| --- \| --- \| \|  \|  \| |

**F.2.1.** Do you have any tinned or jarred fruit in your home now?

Yes □ No □

**F.2.2.** If yes, what types of tinned or jarred fruit do you have in your home now?

This is an open question. As the participant lists the tinned or jarred fruit they have, tick the matching options in the table or add any other tinned or jarred fruit to the free entry box which says other.

When the participant finishes, prompt her/him by reminding her/him of places she/he may have forgotten: Have you remembered tinned or jarred fruit in your fridge and in your cupboards?

| Tins / jars of fruit |
| --- |
| List of standard fruits to choose from as well as a free-entry box for less common items.   \|  \| Yes/ No \| \| --- \| --- \| \| Cherries \|  \| \| Fruit salad/cocktail \|  \| \| Grapefruit \|  \| \| Mandarin orange \|  \| \| Peaches \|  \| \| Pears \|  \| \| Pineapple \|  \| \| Plums \|  \| \| Raspberries \|  \| \| Strawberries \|  \| \| Other \|  \|  \| Other tinned fruit \| Number of other items \| \| --- \| --- \| \|  \|  \| |

**F.3.1.** Do you have any dried fruit, such as raisins, dried apricots, or dates in your home now?

Yes □ No □

**F.3.2.** If yes, what types of dried fruit do you have in your home now?

This is an open question. As the participant lists the dried fruit they have, tick the matching options in the table or add any other dried fruit to the free entry box which says other.

When the participant finishes, prompt her/him by reminding her/him of places she/he may have forgotten: Have you remembered dried fruit in a fruit bowl and in your cupboards?

| Dried fruit |
| --- |
| List of standard fruits to choose from as well as a free-entry box for less common items.   \|  \| Yes/ No \| \| --- \| --- \| \| Apples \|  \| \| Apricots \|  \| \| Banana chips \|  \| \| Currants \|  \| \| Dates \|  \| \| Dried mixed fruit \|  \| \| Prunes \|  \| \| Raisins \|  \| \| Sultanas \|  \|  \| Other dried fruit \| Number of other items \| \| --- \| --- \| \|  \|  \| |

**F.4.1.** Do you have any frozen fruit in your home now?

Yes □ No □

**F.4.2.** If yes, what types of frozen fruit do you have in your home now?

This is an open question. As the participant lists the frozen fruit they have, tick the matching options in the table or add any other frozen fruit to the free entry box which says other.

| Frozen fruit |
| --- |
| List of standard fruits to choose from as well as a free-entry box for less common items.   \|  \| Yes/ No \| \| --- \| --- \| \| Mixed berries \|  \| \| Raspberries \|  \| \| Strawberries \|  \|  \| Other frozen fruit \| Number of other items \| \| --- \| --- \| \|  \|  \| |

**F.5.1.** Would you say that the amount of fruit you currently have in your home is more than usual, less than usual, or about the same?

Less than usual □ The same □ More than usual □

**F.5.2.** Without opening any fridge or cupboard doors, is there any kind of fruit in your home now; displayed out in the open?

A possible response may be that some fruit is behind a door, but it is a glass door and the fruit can be seen. If so, report YES. Another response could be that some fresh fruit is out, but that it is stored very high and can only be viewed with a stool. Is so, report NO.

Yes □ No □

**F.5.3.** Would it be possible for <twin1 name> and <twin2 name> to get any fruit by themselves, without your help? By this, we mean whether it would be physically possible for <twin1 name> and <twin2 name> to get any fruit by themselves, without your help.

Yes □ No □

If different for twins: <twin2 name>: Yes □ No □

If the participant does not indicate whether their response is the same or different for <twin1 name> and <twin2 name>, prompt them to check.

**F.5.4** Are <twin1 name> and <twin2 name> allowed to get any fruit by themselves, without asking you first?

Yes □ No □

If different for twins: <twin2 name>: Yes □ No □

If the participant does not indicate whether their response is the same or different for <twin1 name> and <twin2 name>, prompt them to check.

| **F.6.** On average, how often do <twin1 name> and <twin2 name> eat fruit? This includes fruit that is eaten between meals and fruit that is eaten as part of a meal. Fruit juice is not included.  This is an open question. Do not read the response options aloud but categorize the response accordingly. If the participant does not provide enough information e.g. they may say ‘everyday’, prompt for a fuller response e.g. ‘so is that once a day, 2-3 times a day or 4 or more times a day?’ | | | | | | | | |
| --- | --- | --- | --- | --- | --- | --- | --- | --- |
|  | Never or less than once a month | 1-3 times a month | Once  a week | 2-4 times a week | 5-6 times a week | Once  a day | 2-3 times a day | 4 or more times a day |
| <twin1 name>  <twin2 name> | □ | □ | □ | □ | □ | □ | □ | □ |
|  | □ | □ | □ | □ | □ | □ | □ | □ |

If the participant does not indicate whether their response is the same or different for

<twin1name> and <twin2 name>, prompt them to check.

**Vegetables**

**G.1.1.** Do you have any fresh vegetables in your home now? This includes salad

items such as lettuce, cucumber, and tomato but not potatoes.

Yes □ No □

**G.1.2.** If yes, what types of fresh vegetables do you have in your home now?

This is an open question. As the participant lists the fresh vegetables they have, tick the matching options in the table or add any other fresh vegetables to the free entry box which says other.

When the participant finishes, prompt her/him by reminding her/him of places she/he may have forgotten: Have you remembered fresh vegetables in your fridge and in your cupboards?

| Fresh vegetables |
| --- |
| List of standard vegetables to choose from as well as a free-entry box for less common items.   \|  \| Yes/ No \| \| --- \| --- \| \| Broccoli \|  \| \| Brussel sprouts \|  \| \| Cabbage \|  \| \| Carrots \|  \| \| Cauliflower \|  \| \| Celery \|  \| \| Corn on the cob \|  \| \| Cucumber \|  \| \| Lettuce \|  \| \| Mushrooms \|  \| \| Onions \|  \| \| Peppers \|  \| \| Runner beans/green beans \|  \| \| Swede \|  \| \| Tomatoes \|  \|  \| Other fresh vegetables \| Number of other items \| \| --- \| --- \| \|  \|  \| |

**G.2.1.** Do you have any tinned or jarred vegetables for example tinned tomatoes, sweetcorn, or jarred beetroot, in your home now? This includes tinned pulses such as chickpeas, kidney beans and lentils.

Yes □ No □

**G.2.2.** If yes, what types of tinned or jarred vegetables do you have in your home now?

This is an open question. As the participant lists the tinned or jarred vegetables they have, tick the matching options in the table or add any other tinned or jarred vegetables to the free entry box which says other.

When the participant finishes, prompt her/him by reminding her/him of places she/he may have forgotten: Have you remembered tinned or jarred vegetables in your fridge and in your cupboards?

| Tins of vegetables |
| --- |
| List of standard vegetables to choose from as well as a free-entry box for less common items.   \|  \| Yes/ No \| \| --- \| --- \| \| Baked beans \|  \| \| Bamboo shoots \|  \| \| Beetroot \|  \| \| Broad beans \|  \| \| Carrots \|  \| \| Mixed vegetables \|  \| \| Mushrooms \|  \| \| Peas \|  \| \| Pease pudding \|  \| \| Pickled onion \|  \| \| Pickled gherkins \|  \| \| Runner beans/green beans \|  \| \| Sweetcorn \|  \| \| Tomatoes \|  \|  \| Other tinned vegetables \| Number of other items \| \| --- \| --- \| \|  \|  \| |

**G.3.1.** Do you have any frozen vegetables in your home now?

Yes □ No □

**G.3.2.** If yes, what types of frozen vegetables do you have in your home now?

This is an open question. As the participant lists the frozen vegetables they have, tick the matching options in the table or add any other frozen vegetables to the free entry box which says other.

| Frozen vegetables |
| --- |
| List of standard vegetables to choose from as well as a free-entry box for less common items.   \|  \| Yes/ No \| \| --- \| --- \| \| Broad beans \|  \| \| Brussel sprouts \|  \| \| Cabbage \|  \| \| Cauliflower \|  \| \| Mange tout \|  \| \| Mixed vegetables \|  \| \| Peas \|  \| \| Peppers \|  \| \| Runner beans/green beans \|  \| \| Spinach \|  \| \| Sweet corn \|  \|  \| Other frozen vegetables \| Number of other items \| \| --- \| --- \| \|  \|  \| |

**G.4.1.** Would you say that the amount of vegetables you currently have in your home is more than usual, less than usual, or about the same?

Less than usual □ The same □ More than usual □

**G.4.2.** Do you have any ready to eat fresh vegetables on a shelf in the fridge or on the kitchen counter now? These include baby carrots, cherry tomatoes, or vegetables that you have sliced to make them ready to eat.

Yes □ No □

**G.4.3.** Would it be possible for <twin1 name> and <twin2 name> to get any vegetables by themselves without your help? By this, we mean whether it would be physically possible for <twin1 name> and <twin2 name> to get any vegetables by themselves, without your help.

Yes □ No □

If different for twins: <twin2 name>: Yes □ No □

If the participant does not indicate whether their response is the same or different for <twin1 name> and <twin2 name>, prompt them to check.

**G.4.4.** Are <twin1 name> and <twin2 name> allowed to get any vegetables by themselves, without asking you first?

Yes □ No □

If different for twins: <twin2 name>: Yes □ No □

If the participant does not indicate whether their response is the same or different for <twin1 name> and <twin2 name>, prompt them to check.

| **G.5.** On average, how often do <twin1 name> and <twin2 name> eat vegetables? This includes salad items such as cucumber, lettuce and tomato but not potatoes. Vegetables that are eaten between meals and vegetables that are eaten as part of a meal are included.  This is an open question. Do not read the response options aloud but categorize the response accordingly. If the participant does not provide enough information e.g. they may say ‘everyday’, prompt for a fuller response e.g. ‘so is that once a day, 2-3 times a day or 4 or more times a day?’   \|  \| Never or less than once a month \| 1-3 times a month \| Once  a week \| 2-4 times a week \| 5-6 times a week \| Once  a day \| 2-3 times a day \| 4 or more times  a day \| \| --- \| --- \| --- \| --- \| --- \| --- \| --- \| --- \| --- \| \| <twin1 name>  <twin2 name> \| □ \| □ \| □ \| □ \| □ \| □ \| □ \| □ \| \|  \| □ \| □ \| □ \| □ \| □ \| □ \| □ \| □ \|   If the participant does not indicate whether their response is the same or different for  <twin1name> and <twin2 name>, prompt them to check. |
| --- | --- | --- | --- | --- | --- | --- | --- | --- | --- | --- | --- | --- | --- | --- | --- | --- | --- | --- | --- | --- | --- | --- | --- | --- | --- | --- | --- |

**Savoury snacks**

**H.1.1.** Do you have any savoury snacks for example peanuts, crisps, tortillas and cheesy biscuits in your home now?

Yes □ No □

**H.1.2.** If yes, what types of savoury snacks do you have in your home now? Snacks like plain rice cakes, oatcakes, and breadsticks are not included.

This is an open question. As the participant lists the savoury snacks they have, tick the matching options in the table or add any other savoury snacks to the free entry box which says other.

When the participant finishes, prompt her/him by reminding her/him of places she/he may have forgotten: Have you remembered savoury snacks in your fridge and in your cupboards?

| Savoury snacks |
| --- |
| List of standard Savoury snacks to choose from as well as a free-entry box for less common items.   \|  \| Yes/ No \| \| --- \| --- \| \| Cheese biscuits \|  \| \| Cheese straws \|  \| \| Crisps \|  \| \| Peanuts \|  \| \| Tortilla chips \|  \|  \| Other savoury snacks \| Number of other items \| \| --- \| --- \| \|  \|  \| |

**H.1.3.** Would you say that the amount of savoury snacks you currently have in your home is more than usual, less than usual, or about the same?

Less than usual □ The same □ More than usual □

**H.2.1.** Without opening any fridge or cupboard doors, are there any kind of savoury snacks in your home now; displayed out in the open?

A possible response may be that some savoury snacks are behind a door, but it is a glass door and the snacks can be seen. If so, report YES. Another response could be that some savoury snacks are out, but that they are stored very high and can only be viewed with a stool. Is so, report NO.

Yes □ No □

**H.2.2.** Would it be possible for <twin1 name> and <twin2 name> to get any savoury snacks by themselves, without your help? By this, we mean whether it would be physically possible for <twin1 name> and <twin2 name> to get any savoury snacks by themselves, without your help.

Yes □ No □

If different for twins: <twin2 name>: Yes □ No □

If the participant does not indicate whether their response is the same or different for <twin1 name> and <twin2 name>, prompt them to check.

**H.2.3.** Are <twin1 name> and <twin2 name> allowed to get any savoury snacks by themselves, without asking you first?

Yes □ No □

If different for twins: <twin2 name>: Yes □ No □

If the participant does not indicate whether their response is the same or different for <twin1 name> and <twin2 name>, prompt them to check.

| **H.2.5.** On average, how often do <twin1 name> and <twin2 name> eat savoury snacks such as peanuts, crisps, tortillas and cheesy biscuits? This includes savoury snacks that are eaten between meals and savoury snacks that are eaten as part of a meal such as crisps with lunch. This is an open question. Do not read the response options aloud but categorize the response accordingly. If the participant does not provide enough information e.g. they may say ‘everyday’, prompt for a fuller response e.g. ‘so is that once a day, 2-3 times a day or 4 or more times a day?’ | | | | | | | | | |
| --- | --- | --- | --- | --- | --- | --- | --- | --- | --- |
|  | Never or less than once a month | 1-3 times a month | Once  a week | 2-4 times a week | 5-6 times a week | Once  a day | 2-3 times a day | 4 or more times a day | |
| <twin1 name>  <twin2 name> | □ | □ | □ | □ | □ | □ | □ | □ | |
|  | □ | □ | □ | □ | □ | □ | □ | □ | |

If the participant does not indicate whether their response is the same or different for

<twin1name> and <twin2 name>, prompt them to check.

Any comments about savoury snacks

**Sweet snacks**

**I.3.1.** Do you have any sweet snacks for example cakes, biscuits or ice-cream in your home now?

Yes □ No □

**I.3.2.** If yes, what types of sweet snacks do you have in your home now? Do not include sweets or chocolate.

This is an open question. As the participant lists the sweet snacks they have, tick the matching options in the table or add any other sweet snacks to the free entry box which says other.

When the participant finishes, prompt her/him by reminding her/him of places she/he may have forgotten: Have you remembered sweet snacks in your fridge and in your cupboards?

| Sweet snacks |
| --- |
| List of standard sweet snacks to choose from as well as a free-entry box for less common items.   \|  \| Yes/ No \| \| --- \| --- \| \| Biscuits \|  \| \| Buns \|  \| \| Cakes \|  \| \| Ice-cream \|  \| \| Ice-lollies \|  \| \| Pastries \|  \|  \| Other sweet snacks \| Number of other items \| \| --- \| --- \| \|  \|  \| |

**I.3.3.** Would you say that the amount of sweet snacks you currently have in your home is more than usual, less than usual, or about the same?

Less than usual □ The same □ More than usual □

**I.4.1.** Without opening any fridge or cupboard doors, are there any kind of sweet snacks in your home now displayed out in the open?

If yes, check that the participant is referring to snacks like cakes, biscuits and ice cream. A possible response may be that some sweet snacks are behind a door, but it is a glass door and the snacks can be seen. If so, report YES. Another response could be that some sweet snacks are out, but that they are stored very high and can only be viewed with a stool. Is so, report NO.

Yes □ No □

**I.4.2.** Would it be possible for <twin1 name> and <twin2 name> to get any sweet snacks by themselves, without your help? By this, we mean whether it would be physically possible for <twin1 name> and <twin2 name> to get any sweet snacks by themselves, without your help.

Yes □ No □

If different for twins: <twin2 name>: Yes □ No □

If the participant does not indicate whether their response is the same or different for <twin1 name> and <twin2 name>, prompt them to check.

**I.4.3.** Are <twin1 name> and <twin2 name> allowed to get any sweet snacks by themselves, without asking you first?

Yes □ No □

If different for twins: <twin2 name>: Yes □ No □

If the participant does not indicate whether their response is the same or different for <twin1 name> and <twin2 name>, prompt them to check.

| **I.4.5.** On average, how often do <twin1 name> and <twin2 name> eat sweet snacks such as cakes, biscuits, and ice-cream? This includes sweet snacks that are eaten between meals and sweet snacks that are eaten as part of a meal such as ice-cream for dessert. This is an open question. Do not read the response options aloud but categorize the response accordingly. If the participant does not provide enough information e.g. they may say ‘everyday’, prompt for a fuller response e.g. ‘so is that once a day, 2-3 times a day or 4 or more times a day?’ |
| --- |

|  | Never or less than once a month | 1-3 times a month | Once  a week | 2-4 times a week | 5-6 times a week | Once  a day | 2-3 times a day | 4 or more times a day |
| --- | --- | --- | --- | --- | --- | --- | --- | --- |
| <twin1 name>  <twin2 name> | □ | □ | □ | □ | □ | □ | □ | □ |
|  | □ | □ | □ | □ | □ | □ | □ | □ |

If the participant does not indicate whether their response is the same or different for

<twin1name> and <twin2 name>, prompt them to check.

Any comments about sweet snacks

Any commn

**Confectionery**

**J.1.1.** Do you have any confectionery in your home now? This includes sweets and chocolate.

Yes □ No □

**J.1.2.** If yes, what types of confectionery do you have in your home now?

This is an open question. As the participant lists the confectionery they have, tick the matching options in the table or add any other confectionery to the free entry box which says other.

When the participant finishes, prompt her/him by reminding her/him of places she/he may have forgotten: Have you remembered confectionery in your fridge, in a bowl and in your cupboards?

| Confectionery |
| --- |
| List of standard confectionery to choose from as well as a free-entry box for less common items.   \|  \| Yes/ No \| \| --- \| --- \| \| Chocolate \|  \| \| Sweets \|  \|  \| Other confectionery \| Number of other items \| \| --- \| --- \| \|  \|  \| |

**J.1.3.** Would you say that the amount of confectionery you currently have in your home is more than usual, less than usual, or about the same?

Less than usual □ The same □ More than usual □

**J.2.1.** Without opening any fridge or cupboard doors, is there any kind of confectionery in your home now displayed out in the open?

A possible response may be that some confectionery is behind a door, but it is a glass door and the confectionery can be seen. If so, report YES. Another response could be that some confectionery is out, but that it is stored very high and can only be viewed with a stool. Is so, report NO.

Yes □ No □

**J.2.2.** Would it be possible for <twin1 name> and <twin2 name> to get any confectionery by themselves, without your help? By this, we mean whether it would be physically possible for <twin1 name> and <twin2 name> to get any confectionery by themselves, without your help.

Yes □ No □

If different for twins: <twin2 name>: Yes □ No □

If the participant does not indicate whether their response is the same or different for <twin1 name> and <twin2 name>, prompt them to check.

**J.2.3.** Are <twin1 name> and <twin2 name> allowed to get any confectionery by themselves, without asking you first?

Yes □ No □

If different for twins: <twin2 name>: Yes □ No □

If the participant does not indicate whether their response is the same or different for <twin1 name> and <twin2 name>, prompt them to check.

| **J.2.5.** On average, how often do <twin1 name> and <twin2 name> eat confectionery such as chocolate and fruit sweets? This is an open question. Do not read the response options aloud but categorize the response accordingly. If the participant does not provide enough information e.g. they may say ‘everyday’, prompt for a fuller response e.g. ‘so is that once a day, 2-3 times a day or 4 or more times a day?’ |
| --- |

|  | Never or less than once a month | 1-3 times a month | Once  a week | 2-4 times a week | 5-6 times a week | Once  a day | 2-3 times a day | 4 or more times a day |
| --- | --- | --- | --- | --- | --- | --- | --- | --- |
| <twin1 name>  <twin2 name> | □ | □ | □ | □ | □ | □ | □ | □ |
|  | □ | □ | □ | □ | □ | □ | □ | □ |

If the participant does not indicate whether their response is the same or different for

<twin1name> and <twin2 name>, prompt them to check.

**Section K – FAST FOOD**

| **K.1.1.** On average, how often do <twin1 name> and <twin2 name> eat fast food from places such as McDonald’s, KFC, Burger King, and Subway…? This includes both eating in and taking food away from fast food places. This is an open question. Do not read the response options aloud but categorize the response accordingly. If the participant does not provide enough information e.g. they may say ‘everyday’, prompt for a fuller response e.g. ‘so is that once a day, 2-3 times a day or 4 or more times a day?’ |
| --- |

|  | Never or less than once a month | 1-3 times a month | Once  a week | 2-4 times a week | 5-6 times a week | Once  a day | 2-3 times a day | 4 or more times a day |
| --- | --- | --- | --- | --- | --- | --- | --- | --- |
| <twin1 name>  <twin2 name> | □ | □ | □ | □ | □ | □ | □ | □ |
|  | □ | □ | □ | □ | □ | □ | □ | □ |

If the participant does not indicate whether their response is the same or different for

<twin1name> and <twin2 name>, prompt them to check.

| **K.1.2.** On average, how often do <twin1 name> and <twin2 name> eat other convenience foods for his/her main meal? This includes food that requires no preparation such as ready-made pizza, microwaveable meals, and takeaway food such as fish and chips, Chinese, and Indian… Other convenience food such as fish fingers and chicken nuggets are included. This is an open question. Do not read the response options aloud but categorize the response accordingly. If the participant does not provide enough information e.g. they may say ‘everyday’, prompt for a fuller response e.g. ‘so is that once a day, 2-3 times a day or 4 or more times a day?’ | | | | | | | | |  |
| --- | --- | --- | --- | --- | --- | --- | --- | --- | --- |
|  | Never or less than once a month | 1-3 times a month | Once  a week | 2-4 times a week | 5-6 times a week | Once  a day | 2-3 times a day | 4 or more times a day | |
| <twin1 name>  <twin2 name> | □ | □ | □ | □ | □ | □ | □ | □ | |
|  | □ | □ | □ | □ | □ | □ | □ | □ | |

If the participant does not indicate whether their response is the same or different for

<twin1name> and <twin2 name>, prompt them to check.

**Section L - DRINKS**

**L.1.1.** Do you have any *non-alcoholic* drinks other than water in your home now? Examples are fruit juice, squash, fizzy pop, ready-made fruit flavoured drinks, smoothies, and milk.

*If no to L1.1, skip L1.2. and L1.4. – L2.1. (but do ask L1.3. and L2.2.)*

Yes □ No □

**L.1.2.** If yes, what types of non-alcoholic drinks do you have in your home now?

This is an open question. As the participant lists the drinks they have, tick the matching options in the table. May need to prompt to determine whether each drink is sugar sweetened or not.

When the participant finishes, prompt her/him by reminding her/him of places she/he may have forgotten: Have you remembered non-alcoholic drinks in your fridge and in your cupboards?

|  | | Sugar sweetened:  (Yes/ No) | | Pure juice/No added sugar/diet:  (Yes/ No) | |
| --- | --- | --- | --- | --- | --- |
| Fruit juice e.g. orange, apple | |  | |  | |
| Squash/cordial e.g. Robinson’s blackcurrant cordial | |  | |  | |
| Fizzy pop e.g. coke, lemonade | |  | |  | |
| Ready made fruit flavoured drinks e.g. Ribena, Oasis | |  | |  | |
| Smoothies | |  | |  | |
|  | Skimmed:  (yes/ no) | | Semi-skimmed:  (yes/ no) | | Full-fat:  (yes/ no) |
| Milk |  | |  | |  |

**L.1.3.** Would you say that the amount of non-alcoholic drinks you currently have in your home is more than usual, less than usual, or about the same?

Less than usual □ The same □ More than usual □

**L.1.4.** Without opening any fridge or cupboard doors, are there any non-alcoholic drinks in your home now; displayed out in the open?

A possible response may be that some drinks are behind a door, but it is a glass door and the drinks can be seen. If so, report YES. Another response could be that some drinks are out, but that they are stored very high and can only be viewed with a stool. Is so, report NO.

Yes □ No □

**L.1.5.** If yes, what types of non-alcoholic drinks are displayed out in the open?

This is an open question. As the participant lists the drinks they have, tick the matching drink type in the table. May need to prompt the participant to determine whether each drink is sugar sweetened or not. For example, if the participant just says ‘coke’ interviewer says ‘is that diet coke?’ If the participant just says ‘orange juice’ interviewer says ‘is that with added sugar?’ etc.

|  | | Sugar sweetened:  (yes/ no) | | Pure juice/No added sugar/diet:  (yes/ no) | |
| --- | --- | --- | --- | --- | --- |
| Fruit juice e.g. orange, apple | |  | |  | |
| Squash/cordial e.g. Robinson’s blackcurrant cordial | |  | |  | |
| Fizzy pop e.g. coke, lemonade | |  | |  | |
| Ready made fruit flavoured drinks e.g. Ribena, Oasis | |  | |  | |
| Smoothies | |  | |  | |
|  | Skimmed:  (yes/ no) | | Semi-skimmed:  (yes/ no) | | Full-fat:  (yes/ no) |
| Milk |  | |  | |  |

**L.1.6.** Would it be possible for <twin1 name> and <twin2 name> to get any drinks by themselves, without your help? By this, we mean whether it would be physically possible for <child’s name> to get any drinks by him/herself, without your help.

We are referring to non-alcoholic drinks other than water.

Yes □ No □ *If no, skip L1.7 – L1.9.*

If different for twins: <twin2 name>: Yes □ No □

If the participant does not indicate whether their response is the same or different for <twin1 name> and <twin2 name>, prompt them to check.

**L.1.7.** If yes, what types of drinks could <twin1 name> and <twin2 name> get by themselves, without your help?

We are referring to non-alcoholic drinks other than water.

This is an open question. As the participant lists the drinks they have, tick the matching drink type in the table. May need to prompt the participant to determine whether each drink is sugar sweetened or not.

|  | | Sugar sweetened:  (yes/ no) | | Pure juice/No added sugar/diet:  (yes/ no) | |
| --- | --- | --- | --- | --- | --- |
| Fruit juice e.g. orange, apple | |  | |  | |
| Squash/cordial e.g. Robinson’s blackcurrant cordial | |  | |  | |
| Fizzy pop e.g. coke, lemonade | |  | |  | |
| Ready made fruit flavoured drinks e.g. Ribena, Oasis | |  | |  | |
| Smoothies | |  | |  | |
|  | Skimmed:  (yes/ no) | | Semi-skimmed:  (yes/ no) | | Full-fat:  (yes/ no) |
| Milk |  | |  | |  |

**L.1.8.** Are <twin1 name> and <twin2 name> allowed to get any drinks by themselves, without asking you first? We are referring to non-alcoholic drinks other than water.

Yes □ No □

If different for twins: <twin2 name>: Yes □ No □

If the participant does not indicate whether their response is the same or different for <twin1 name> and <twin2 name>, prompt them to check.

**L.1.9.** If yes, what types of drinks are <twin1 name> and <twin2 name> allowed to get by themselves, without asking you first?

We are referring to non-alcoholic drinks other than water. This is an open question. As the participant lists the drinks they have, tick the matching drink type in the table. May need to prompt the participant to determine whether each drink is sugar sweetened or not.

|  | | Sugar sweetened:  (yes/ no) | | Pure juice/No added sugar/diet:  (yes/ no) | |
| --- | --- | --- | --- | --- | --- |
| Fruit juice e.g. orange, apple | |  | |  | |
| Squash/cordial e.g. Robinson’s blackcurrant cordial | |  | |  | |
| Fizzy pop e.g. coke, lemonade | |  | |  | |
| Ready made fruit flavoured drinks e.g. Ribena, Oasis | |  | |  | |
| Smoothies | |  | |  | |
|  | Skimmed:  (yes/no) | | Semi-skimmed:  (yes/ no) | | Full-fat:  (yes/ no) |
| Milk |  | |  | |  |

| **L.2.** On average, how often do <twin1 name> and <twin2 name> drink… Read each drink type (with examples) in turn and wait for the participant’s response before moving onto the next drink type. Ask for all drinks whether they are in the home or not. This is an open question. Do not read the response options aloud but categorize the response accordingly. If the participant does not provide enough information e.g. they may say ‘everyday’, the interviewer should prompt for a fuller response e.g. ‘so is that once a day, 2-3 times a day or 4 or more times a day?’ | | | | | | | | | |
| --- | --- | --- | --- | --- | --- | --- | --- | --- | --- |
|  | | Never or less than once a month | 1-3 times a month | Once  a week | 2-4 times a week | 5-6 times a week | Once  a day | 2-3 times a day | 4 or more times a day |
| Sugar-sweetened drinks such as original coke, squash with sugar, or ready-made fruit flavoured drinks with sugar such as original ribena or fruit shoots. | <twin1 name> | □ | □ | □ | □ | □ | □ | □ | □ |
|  | <twin2 name> | □ | □ | □ | □ | □ | □ | □ | □ |

| Sugar-free drinks such as diet coke, squash with no added sugar, or ready-made fruit flavoured drinks with no added sugar such as ribena light or fruit shoots with low sugar. | <twin1 name> | □ | □ | □ | □ | □ | □ | □ | □ |
| --- | --- | --- | --- | --- | --- | --- | --- | --- | --- |
|  | <twin2 name> | □ | □ | □ | □ | □ | □ | □ | □ |

| \| Fruit juice such as orange or apple juice. \| <twin1 name> \| □ \| □ \| □ \| □ \| □ \| □ \| □ \| □ \| \| --- \| --- \| --- \| --- \| --- \| --- \| --- \| --- \| --- \| --- \| \|  \| <twin2 name> \| □ \| □ \| □ \| □ \| □ \| □ \| □ \| □ \| |
| --- | --- | --- | --- | --- | --- | --- | --- | --- | --- | --- | --- | --- | --- | --- | --- | --- | --- | --- | --- | --- |

| Milk (this includes milk on cereal). | <twin1 name> | □ | □ | □ | □ | □ | □ | □ | □ |
| --- | --- | --- | --- | --- | --- | --- | --- | --- | --- |
|  | <twin2 name> | □ | □ | □ | □ | □ | □ | □ | □ |

**Section M – MEALTIMES**

**M1.3.** How many days a week do your family sit at a table to eat breakfast together? This includes occasions when it is just <twin1 name> and <twin2 name> and yourself or just <twin1 name> and <twin2 name> and your <husband/wife/partner>. Only include occasions where you or your <husband/wife/partner> actually eat with your twins.

A possible response might be that they sit down as a family to eat breakfast, but not at a dining table. This is not included. Another possible response is that the twins sit at a table to eat breakfast with their siblings, but not their parent(s). This is not included.

0 1 2 3 4 5 6 7 (days a week)

If different for twins: <twin2 name>: 0 1 2 3 4 5 6 7

If the participant does not indicate whether their response is the same or different for <twin1 name> and <twin2 name>, prompt them to check.

**M2.3.** How many days a week do your family sit at a table to eat a midday meal together?

This includes occasions when it is just <twin1 name> and <twin2 name> and yourself or just <twin1 name> and <twin2 name> and your <husband/wife/partner>. Only include occasions where you or your <husband/wife/partner> actually eat with your twins.

A possible response might be that they sit down as a family to eat a midday meal, but not at a dining table. This is not included. Another possible response is that the twins sit at a table to eat a midday meal with their siblings, but not their parent(s). This is not included.

0 1 2 3 4 5 6 7 (days a week)

If different for twins: <twin2 name>: 0 1 2 3 4 5 6 7

If the participant does not indicate whether their response is the same or different for <twin1 name> and <twin2 name>, prompt them to check.

**M3.3.** How many days a week do your family sit at a table to eat an evening meal together? This includes occasions when it is just <twin1 name> and <twin2 name> and yourself or just <twin1 name> and <twin2 name> and your <husband/wife/partner>. Only include occasions where you or your <husband/wife/partner> actually eat with your twins.

A possible response might be that they sit down as a family to eat an evening meal, but not at a dining table. This is not included. Another possible response is that the twins sit at a table to eat an evening meal with their siblings, but not their parent(s). This is not included.

0 1 2 3 4 5 6 7 (days a week)

If different for twins: <twin2 name>: 0 1 2 3 4 5 6 7

If the participant does not indicate whether their response is the same or different for <twin1 name> and <twin2 name>, prompt them to check.

**Section N – FOOD SHOPPING**

**N1.1.** How often do you shop for food?

This is an open question. Do not read the response options aloud but categorize the response accordingly. If the participant does not provide enough information the interviewer should prompt for a fuller response. For example, if the participant says ‘monthly big trip’, the interviewer should say ‘so is that with few small trips or no small trips?’ If participants say they do online shopping, also categorize their response according to the following options.

□ Monthly, big trip, no small trips

□ Monthly, big trip, few small trips

□ Every other week, big trip, no small trips

□ Every other week, big trip, few small trips

□ Weekly, big trip, no small trips

□ Weekly, big trip, few small trips

□ As and when, no big trip, all small trips as needed

□ Twice each week, big trips, no small trips

□ Twice each week, big trips, few small trips

**N1.2.** How often do <twin1 name> and <twin2 name> go food shopping with you? Please choose a score from 1 to 5: 1 means never, 2 means rarely, 3 means some of the time, 4 means most of the time, 5 means all of the time?

Participants may respond before you get a chance to read them the options. Let them finish and then say, ‘ok, can you tell me whether this happens 1 never, 2 rarely…etc.’

1 2 3 4 5 (5=all of the time)

If different arrangement for twins: <twin2 name>: 1 2 3 4 5

**N1.3.** How many days has it been since you last shopped for food?

If participants only do online shopping, which is then delivered, make sure they are asked about how many days it has been since food was last *delivered* to their house. Participants may say that they have done their next online shop recently but we want to know how many days it has been since food shopping came into the house.

…… days

**N1.4.** Was the last shop small or big?

Small □ Big □ Medium □

**Section O – Height and Weight**

Finally, the last few questions are about your twin’s growth.

**O1.** Do you have any recent height or weight measurements for <twin1 name> and <twin2 name>?

The most recent measurements we have were taken on …...

Yes □ No □

**O2.** Would now be a convenient time to take the twins’ height and weight measurements?

If yes, Using the height chart and scales we have sent, please can you take today’s heights and weights for each of the twins and then read them out to me? Please remember to measure and weigh the twins in indoor clothes without shoes.

Once the twins have been measured and weighed add the measurements to the table at the bottom of the page.

If no, Would you be able to take these measurements tomorrow?

If yes, Please use the height chart and scales we have sent to take the twins heights and weights. Remember to measure and weigh the twins in indoor clothes without shoes. Once you have taken these measurements, please send them to us by email, give them over the telephone, or add them on the Gemini website.

How would you like to give these measurements?

If email, please email to [Gemini@.ucl.ac.uk](mailto:Gemini@.ucl.ac.uk)

(make sure participant includes their Gemini ID number and the date the measurements were taken).

If telephone, please call 020 7679 1723.

If Gemini website, please go to [www.attitudestohealth.co.uk/gemini](http://www.attitudestohealth.co.uk/gemini) and click where it says enter height/weight measurements.

If no, when would be a convenient time for you to take these measurements? Repeat the text beneath tomorrow’s measurements, making sure you record how participants will give the measurements and when they will give them.

| **Twin ID** | **Date measured** | **Height** | **Weight** |
| --- | --- | --- | --- |
|  |  |  |  |
|  |  |  |  |
|  |  |  |  |
|  |  |  |  |

That’s the end of the interview now. Thank you very much for your time. Do you have any questions or comments?

| Add any comments here. |
| --- |
|  |
